# Enhanced characterization of calcified areas in intravascular ultrasound virtual histology images by quantification of the acoustic shadow: validation against computed tomography coronary angiography

**DOI:** 10.1007/s10554-015-0820-x

**Published:** 2015-12-14

**Authors:** Alexander Broersen, Michiel A. de Graaf, Jeroen Eggermont, Ron Wolterbeek, Pieter H. Kitslaar, Jouke Dijkstra, Jeroen J. Bax, Johan H. C. Reiber, Arthur J. Scholte

**Affiliations:** Division of Image Processing, Department of Radiology, Leiden University Medical Center, Leiden, The Netherlands; Department of Cardiology, Leiden University Medical Center, Albinusdreef 2, Postal zone 2300 RC, 2333 ZA Leiden, The Netherlands; The Interuniversity Cardiology Institute of the Netherlands, Utrecht, The Netherlands; Department of Medical Statistics and Bioinformatics, Leiden University Medical Center, Leiden, The Netherlands; Medis Medical Imaging Systems bv, Leiden, The Netherlands

**Keywords:** Quantitative CT angiography, Plaque constitution, Tissue characterization, Acoustic shadow

## Abstract

We enhance intravascular ultrasound virtual histology (VH) tissue characterization by fully automatic quantification of the acoustic shadow behind calcified plaque. VH is unable to characterize atherosclerosis located behind calcifications. In this study, the quantified acoustic shadows are considered calcified to approximate the real dense calcium (DC) plaque volume. In total, 57 patients with 108 coronary lesions were included. A novel post-processing step is applied on the VH images to quantify the acoustic shadow and enhance the VH results. The VH and enhanced VH results are compared to quantitative computed tomography angiography (QTA) plaque characterization as reference standard. The correlation of the plaque types between enhanced VH and QTA differs significantly from the correlation with unenhanced VH. For DC, the correlation improved from 0.733 to 0.818. Instead of an underestimation of DC in VH with a bias of 8.5 mm^3^, there was a smaller overestimation of 1.1 mm^3^ in the enhanced VH. Although tissue characterization within the acoustic shadow in VH is difficult, the novel algorithm improved the DC tissue characterization. This algorithm contributes to accurate assessment of calcium on VH and could be applied in clinical studies.

## Introduction

Intravascular ultrasound virtual histology™ (IVUS VH) is considered to be the gold standard for in vivo assessment of coronary plaque characteristics [1, 2]. However, a limitation of this technique is the ability of the ultrasound signal to fully penetrate calcified tissue, resulting in an acoustic shadow behind calcified tissue [3]. Therefore, IVUS only visualizes the leading edge of calcium, due to a nearly total reflection of the echo signal. Moreover, tissue located behind large calcifications with a clearly edged acoustic shadow cannot be classified accurately and determination of the extent of the calcifications and other plaque characteristics is not possible [4]. Even though the accuracy is unknown, IVUS based tissue characterization methods such as VH^®^ (Volcano) or iMap™ (Boston Scientific) [5] provide classifications of the plaque in these acoustic shadow areas. However, coronary atherosclerosis in the acoustic shadow is rarely classified as dense calcium (DC). This may lead to underestimating the DC area and overestimating of other plaque components. We hypothesized that most of the tissue in the acoustic shadow is DC and aim to compensate for the underestimated calcified areas in VH.

Therefore, a novel masking algorithm was developed in which a fully automatic post-processing step is applied on the VH images to quantify the acoustic shadow behind calcified areas. The quantified regions are modified and added to the total DC volume. To validate this new post-processing step, the enhanced VH (eVH) results are compared to plaque characteristics obtained with computed tomography angiography (CTA) [6].

## Materials and methods

The study population consisted of a previously described patient cohort of 57 patients with chest pain [6]. In brief, all patients underwent CTA followed by clinically referred invasive coronary angiography (ICA) and IVUS VH. The Institutional Review Board of the Leiden University Medical Center approved this retrospective evaluation of clinically collected data, and waived the need for written informed consent.

### IVUS virtual histology

#### Acquisition and quantification

The examinations were acquired during conventional ICA using a dedicated IVUS console (S5™ Imaging system Volcano Corporation, Rancho Cordova, CA, USA) in combination with a 20 MHz, 2.9 F phased-array IVUS Catheter (Eagle Eye, Volcano Corporation, Rancho Cordova, CA, USA).

All IVUS lumen and vessel wall delineations were quantified with QCU-CMS-Research v4.69 (research version of QIvus, developed by the Leiden University Medical Center). Additionally, the tissue region between the lumen and vessel wall was characterized by the integrated Volcano VH software in the following plaque types: necrotic core (NC), dense calcium (DC), fibrotic tissue (FI) and fibro-fatty tissue (FF).

#### Post-processing of the acoustic shadow

**Fig. 1:**
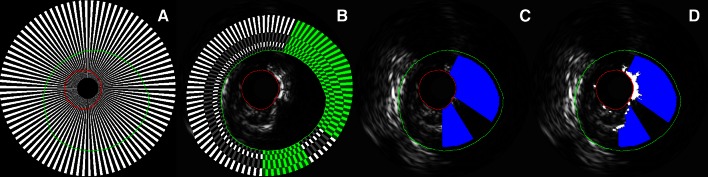
The automatic post-processing steps on the IVUS VH. **a** The first step shows the 180 wedges. **b** Shows the second step with the four predefined layers in *grey* and *white* and in *green* the potential acoustic shadows. **c** Shows the third step with shadows in *blue* where the calcium regions are larger than 20 %. **d** Shows the final masks with the calcified regions in *white*

A novel automatic method was developed to combine VH tissue characterization with an acoustic shadow detection method in order to quantify calcified plaque behind the acoustic shadow. This is performed in five automatic steps as shown in Fig. 1:

1. The potential acoustic shadow regions are determined for each transversal IVUS frame by dividing it into two-degree wide wedges, resulting in 180 wedges.
2. For every wedge, the mean and maximum grayscale intensities of the plaque area are compared to the corresponding mean and maximum grayscale intensities of four predefined layers located outside of the external elastic membrane (0–0.2, 0.2–0.5, 0.5–1.0 and 1.0–2.0 mm). If the mean and maximum grayscale intensity of each layer is less than the corresponding mean and maximum grayscale intensities of the plaque area, the wedge is marked as potential acoustic shadow.
3. Every transversal frame is analysed per one degree along a virtual polar scan-line. If the sum of grayscale intensities from the lumen border up to and including the VH determined dense calcium region is larger than 20 % of the sum of all grayscale intensity values of the scan-line, the angle is marked as an acoustic shadow angle.
4. A mask is constructed based on the marked acoustic shadow angles covering all pixels behind the dense calcified regions as shown in Fig. 1d.
5. The area and volume of the masked areas are quantified and added to the total DC area and DC volume.

### Computed tomography coronary angiography

#### Acquisition

Of the 57 patients, 16 CTA exams were acquired using a 64-row helical CT scanner (Aquilion 64, Toshiba Medical System, Otawara, Japan) and 41 from a 320-row volumetric scanner (Aquilion ONE, Toshiba Medical System, Otawara, Japan). The scan protocol was previously described [7, 8]. Scans with poor image quality were excluded for the current analysis.

#### Quantification of coronary atherosclerosis

**Fig. 2:**
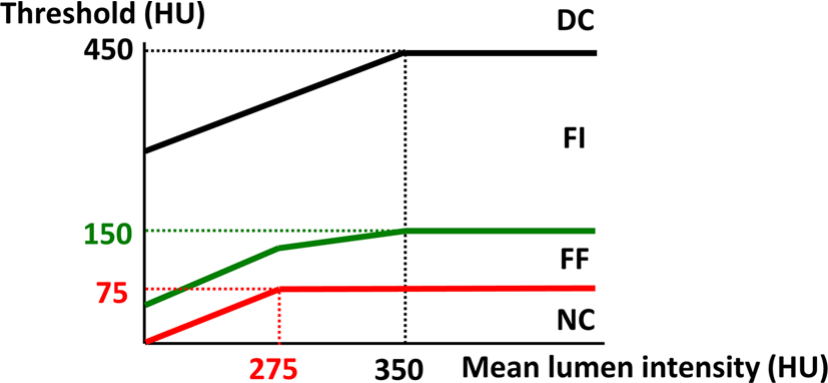
The adaptive threshold scheme. The *graphs* shows the thresholds in HU (above the *black line* is DC, above the *green line* is FI, above the *red line* is FF and below the *red line* is NC) that are used for each measured mean lumen intensity on the X-axis for each cross sectional CTA image. For example, if the mean lumen intensity is more than 350 HU, everything below 75 HU is NC, between 76 and 150 is FF, between 151 and 450 is FI and above 451 is DC

CAD on CTA was quantified using dedicated software (QAngio CT Research Edition v1.3.6, Medis medical imaging systems bv, Leiden, the Netherlands). The validity of this software tool for the segmentation of the coronary anatomy was previously established [9]. For characterization of CAD, two different approaches are available in the software. One approach with predefined fixed intensity cut-off values on the Hounsfield Units (HU) and an adaptive approach where cut-off values are adapted according to lumen attenuation. In the present analysis the adaptive threshold for CTA plaque constitution was used as the reference standard [6]. This adaptive threshold is based on the principle that plaque intensity is influenced by luminal contrast densities and decrease from the proximal to the distal part of the vessel. Therefore, in this automatic- and user-independent approach, the HU cut-off values of the different plaque types are adapted according to lumen intensity. First, a linear trend line is fitted through the mean lumen intensity. Next, the threshold for NC is defined as 200 HU below this estimate with a maximum of 75 HU and the DC threshold is defined as 100 HU above this estimate with a maximum of 450 HU. The threshold between FI and FF is set on 20 % of the difference between the NC and DC threshold. This way, the intensity cut-off values are adapted by the same linear, decreasing trend along the vessel (see Fig. 2). Additionally, because the lumen intensity is lower in parts of a severe stenosis, the NC cut-off value is locally decreased with 125 % of the difference between the estimate and the real lumen intensities. In contrast, lumen intensity is higher in calcified parts due to blooming artefacts. Therefore, the DC cut-off value is locally increased with 25 % of the difference between the estimate and the real lumen intensities.

### Comparison between VH and CTA

**Fig. 3:**
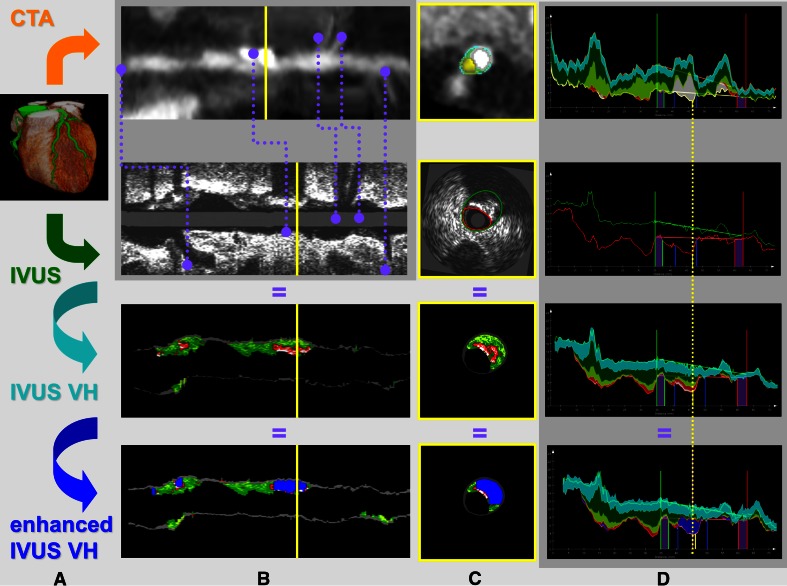
Schematic illustration of the comparison of VH and eVH with QCT. First, the *centerline* was generated from the CTA data set (**a**). IVUS images are fused with the CTA volume using anatomical landmarks (**b**). Lumen and vessel wall contours were detected for both imaging modalities (**c**). Finally, lesions were quantified on the segmented plaque as shown in panel (**d**). Plaque volumes and plaque types were derived for the whole lesion, ranging from the proximal to distal lesion marker (*blue markers*). Both VH and eVH were compared to QCT

**Fig. 4:**
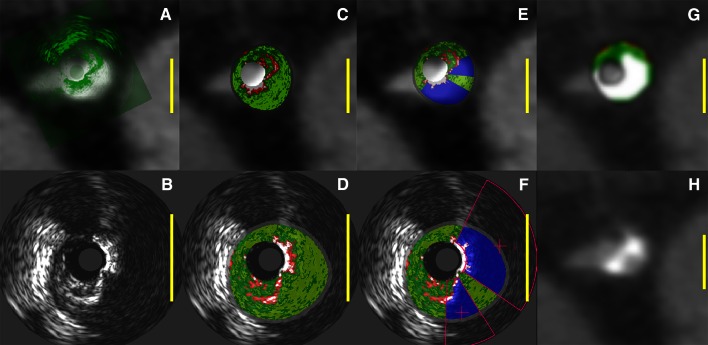
Example of the registered images after fusion. The *yellow scale bars* represent 5 mm. **a** CTA image with IVUS overlay **b** in *green* correctly translated and rotated on the CTA image. **c** Contains the corresponding VH overlay from **d** and similarly **e** contains the eVH image from **f**. The QCT tissue overlay is shown in **g** from the CTA image in **h.** Note that all overlays are mirrored in the *top row* for a correct fusion. The overlays in C–G have five color codes: *red* (NC), *light*-*green* (FF), *dark*-*green* (FI), *white* (DC), and *blue* for the *masked areas*. There is a nice correspondence between the masked areas in **e** and the two calcified areas with high intensities in **h** with in between a calcified area with lower intensity, more similar to the luminal contrast intensity in the bifurcating artery. This example shows that the DC area in VH is underestimated when compared to the DC area in the tissue overlay in **g**. Adding the blue quantified acoustic shadow to the total DC volume in eVH will approximate the DC volume in QCT

To compare the VH data with the CTA data a previously described comparison algorithm was used [6]. All transversal IVUS images were matched and fused with the corresponding transversal CTA images using anatomical landmarks (side-branches, ostia, and calcified plaques) as shown in Fig. 3. The plaque volumes of all different plaque components in the lesions in each corresponding artery were assessed. Both the original VH images and the post-processed, eVH images were compared to QCT as shown in Fig. 4.

### Statistical analysis

Statistical analyses were performed with the use of SPSS software (version 20.0, SPSS Inc., Chicago, IL, USA). First, the absolute plaque volumes of each plaque type on VH and eVH were compared. Second, both the VH and eVH plaque parameters were compared to QCT. For this purpose, the Spearman correlations were calculated. Moreover, the absolute median differences between QCT and VH or eVH were established. Thereafter, Bland–Altman plots were made to assess the bias and the limits of agreement for the comparison between VH and CTA (GraphPad Prism software, version 5.01, San Diego, California, USA). These plots show the difference of each pair plotted against the average value of each pair. Additionally, the Pitman–Morgan test of variances [10] was used to demonstrate if the variances in the comparisons of QCT with VH with and without post-processing were significantly different. The Pitman-Morgan test takes the correlation between two variances into account. A *P* value ≤0.05 was considered statistically significant.

## Results

Baseline patient characteristics were previously described [6]. For this analysis, 109 vessels were used of which 69 revealed atherosclerosis, whereas 40 vessels did not. In these 69 diseased vessels, 108 lesions were identified. These 108 lesions were used for the present lesion based comparison.

### Plaque classification on VH compared to eVH

The quantification results of both VH and eVH are depicted in Table 1. On VH median FI volume was 39.7 (19.9–67.3) mm^3^, while applying the novel algorithm decreased the median FI volume to 37.6 (16.8–61.5) mm^3^, (*p* < 0.001). Similarly, the total volume of FF decreased from 9.3 (4.9–19.4) mm^3^ to 7.9 (4.1–16.9) mm^3^, (*p* < 0.001). Also, NC volume decreased from 11.8 (6.0–22.3) mm^3^ to 10.1 (4.2–18.8) mm^3^, (*p* < 0.001).

**Table 1:** Absolute differences between VH versus eVH plaque constitution compared to QCT plaque constitution (n = 108)

	VH median (IQR) (mm^3^)	QCT median (IQR) (mm^3^)	95 % CI of mean differences	*p* value
Fibrotic	39.7 (19.9–67.3)	55.7 (36.1–94.9)	15.0; 25.9	<0.001
Enhanced fibrotic	37.6 (16.8–61.5)		18.5; 30.2	<0.001
Fibro fatty	9.3 (4.9–19.4)	28.3 (16.2–45.9)	16.7; 23.6	<0.001
Enhanced fibro fatty	7.9 (4.1–16.9)		19.4; 25.9	<0.001
Necrotic core	11.8 (6.0–22.3)	11.0 (5.6–24.7)	−1.6; 3.6	0.458
Enhanced necrotic core	10.1 (4.2–18.8)		1.1; 6.3	0.006
Dense calcium	5.4 (1.7–11.6)	6.95 (0.9–18.9)	4.5; 12.5	<0.001
Enhanced dense calcium	10.0 (3.0–22.8)		−3.6; 1.5	0.401

*eHV* enhanced virtual histology, *CI* confidence interval, *IQR* interquartile range, *VH* virtual histology, *QCT* quantitative computed tomography

In the total population, the volume of DC in VH was 815.22 mm^3^. An acoustic shadow was detected in 106 of the 108 (98 %) lesions. The quantified acoustic shadow resulted in a total volume of 1033.93 mm^3^. These quantified areas were added to the DC volumes providing a total enhanced DC volume of 1949.15 mm^3^. Overall, the median DC volume increased from 5.4 (1.7–11.6) mm^3^ to 10.0 (3.0–22.8) mm^3^, (*p* < 0.001).

### Plaque classification on QCT

The QCT plaque quantification results were previously described [6]. In brief, on QCT, the median FI volume was 55.7 (36.1–94.9) mm^3^, the median FF volume was 28.3 (16.2–45.9) mm^3^, the median volume of NC was 11.0 (5.6–24.7) mm^3^ and the DC volume was 6.95 (0.9–18.9) mm^3^.

### Comparison of VH and eVH to QCT plaque classification

To validate the novel algorithm, the VH and eVH data were compared to QCT. The results of this comparison are demonstrated in Table 1.

#### Absolute differences

The median DC volume was significantly underestimated by VH compared to QCT (5.4 (1.7–11.6) mm^3^, vs. 6.95 (0.9–18.9) mm^3^, *p* < 0.001). After applying the novel algorithm to the VH data, there was no significant difference between the DC volume on both modalities 10.0 (3.0–22.8) mm^3^ for eVH versus 6.95 (0.9–18.9) mm^3^ for QCT, *p* = 0.401). However, for NC a significant difference between QCT and eVH was observed (11.0 (5.6–24.7) mm^3^ on QCT vs 10.1 (4.2–18.8) mm^3^ on eVH, *p* = 0.006). For FI and FF a significant difference was observed for both VH and eVH compared to QCT.

#### Correlation and agreement

For all four plaque types there was difference in correlation between QCT and VH compared to QCT and eVH after applying the novel algorithm (Table 2). The largest change in correlation between VH and QCT was observed for DC. Adding the quantified area to the DC volume improved the correlation with QCT from 0.733 to 0.818. The correlations of the other plaque types changed less if the novel algorithm was applied to the VH data.

**Table 2:** Correlation and agreement of VH versus eVH plaque constitution compared to QCT plaque constitution (n = 108)

	Correlation (Spearman)	Bias (mm^3^)	Lower 95 % LOA (mm^3^)	Upper 95 % LOA (mm^3^)	Difference in variance (*p* values Pitman’s test)
Fibrotic	0.787, <0.001	20.4	−35.7	76.6	<0.01
Enhanced fibrotic	0.750, <0.001	24.3	−54.6	77.8	
Fibro fatty	0.704, <0.001	20.2	−15.2	55.6	0.05
Enhanced fibro fatty	0.728, <0.001	22.6	−7.3	44.5	
Necrotic core	0.479, <0.001	1.0	−25.9	27.9	0.76
Enhanced necrotic core	0.425, <0.001	3.7	−26.4	61.4	
Dense calcium	0.733, <0.001	8.5	−32.5	49.5	<0.001
Enhanced dense calcium	0.818, <0.001	−1.1	−28.3	31.7	

*VH* virtual histology, *LOA* limits of agreement, *QCT* quantitative computed tomography

**Fig. 5:**
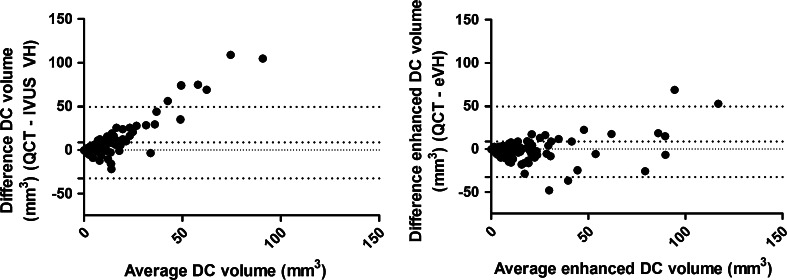
Bland–Altman plots for both the DC volume of VH and DC volume of eVH compared with QCT

**Fig. 6:**
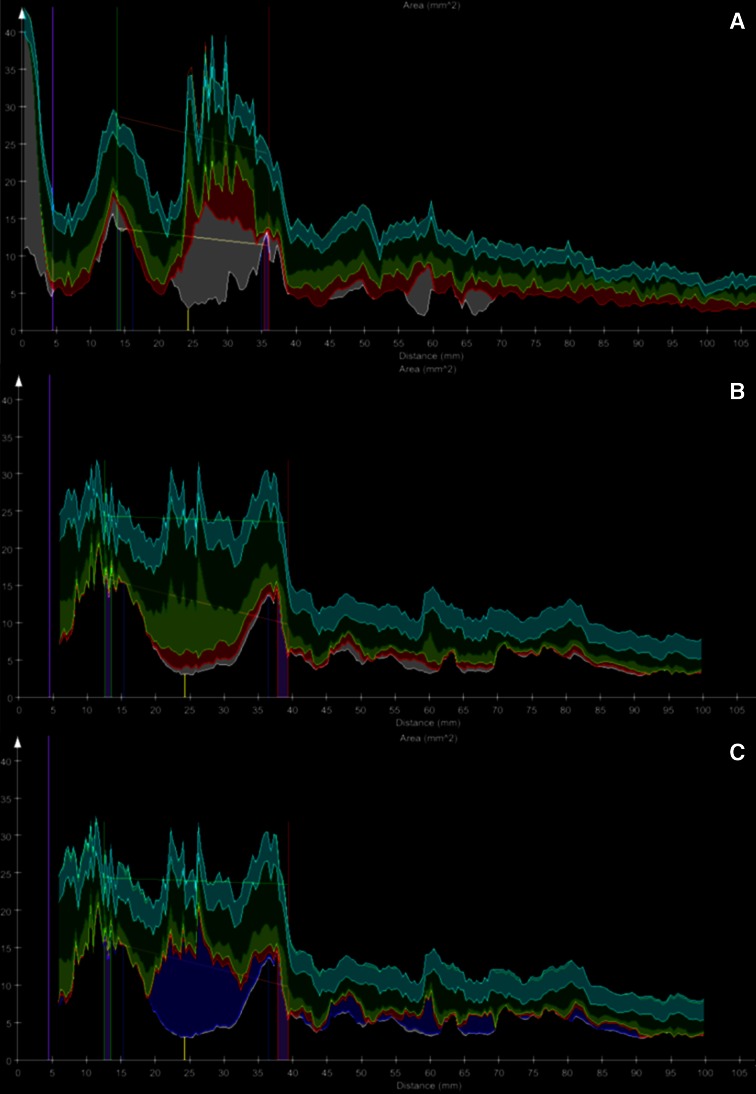
Quantification of plaque volumes along the vessel. **a** An example of a coronary lesion in CTA. The X-axis represents the distance from the coronary ostium in mm. The Y-axis represents the area of either the lumen (*lower part of graph*) or the vessel wall (*upper part of graph*) in mm^2^. The part between the two graphs shows the plaque constitution using a *color code*. In the corresponding VH data is shown in **b** and in **c** the results with the quantified *shadows in blue*. The enhanced quantification has better correspondence with the CTA analysis after adding the quantified *shadow areas* to the DC

The results of the corresponding Bland–Altman plots for the DC volume of both VH and eVH compared to QCT are shown in Table 2 and depicted in Fig. 5. Without masking, DC volume in VH was underestimated with a bias of 8.5 mm^3^ compared to the QCT. After applying the masking tool it was overestimated with a bias of −1.1 mm^3^. The masking is especially useful on cases with large DC volumes as shown in Fig. 5 where the systematic error in the large DC volumes is smaller for eVH. The last column in Table 2 depicts the statistical significance of the difference in variances between the two comparisons (i.e. QCT vs. VH and QCT vs. eVH). As demonstrated with the Pitman-Morgan test of variances, the agreement in DC volume between VH and QCT was significantly improved by applying the masking tool (*p* < 0.001). Similarly, there was a significant change in variance for the FF and FI between VH and eVH compared to QCT. However, for these plaque types the agreement of QCT was better with VH than with eVH. For NC, there was no significant difference in variances between QCT versus VH and QCT versus enhanced VH. An example of a coronary lesion and the resulting comparison between QCT and VH without and with the enhanced plaque quantification is shown in Fig. 6.

## Discussion

This study presents the results of a novel post-processing step on VH data to compensate for the limited ability of IVUS to penetrate dense calcified tissue. The novel algorithm detects, masks, and quantifies the acoustic shadow behind calcium. By adding the quantified acoustic shadows to the total DC volume, the correlation between DC volumes on VH and QCT improves significantly. Moreover, the agreement between both modalities improved significantly, from an underestimation in VH to a small overestimation of DC volume in the enhanced VH. However, for FI and FF the agreement with QCT was reduced after applying the novel algorithm.

### Acoustic shadow

The limited ability of the echo-signal to penetrate coronary calcium results in two problems [5]. Firstly, the outer vessel boundaries located in the acoustic shadow are difficult to segment and need to be manually adjusted. This potentially leads to observer bias. However, an experienced observer can manually overcome this problem. Secondly and most important, the noise in the acoustic shadow is classified as coronary atherosclerosis by RF based methods as VH or iMap™(Boston Scientific) [11]. Ideally, an acoustic shadow should be completely dark in B-mode IVUS images due to the greatly limited ability of the ultrasound signal to penetrate the calcified plaque. Current software tools fail to identify these acoustic shadows and quantify the tissue within the acoustic shadow. These regions are characterized mainly as FI or FF by VH or characterized mainly as necrotic tissue by iMap. The validity of this quantification is unknown. Recent echogenicity methods [12] solve this problem by classifying the acoustic shadows behind calcifications as ‘unknown’. The acoustic shadow can be delineated manually, but is very time consuming. Moreover, a manual approach is susceptible to inter- and intra-observer variability for determining and masking the regions in the acoustic shadows. Bayturan et al. [13] investigated a novel type of ‘attenuated plaque’ which occurs in the absence of DC. Attenuated plaque was defined as hypoechoic plaque with deep ultrasonic attenuation (shadow) despite the absence of DC and is associated with high risk lesions. The acoustic shadow behind this attenuated plaque can also be detected by this post-processing procedure by using step 4 to differentiate between attenuated shadow regions and shadow regions resulting from DC as classified by VH or for example by echogenicity [14] if there is no VH available.

### Novel algorithm

For the present study a novel algorithm was developed to automatically detect and quantify the regions of acoustic shadow. Our hypothesis is that most of the tissue in the acoustic shadow is DC, or at least a larger volume than is detected with IVUS VH. To compensate for the suspected underestimation of DC within these regions, the quantified areas were added to the total DC volume. Although the corrected DC volumes in eVH show improved bias and correlation with QCT, the post-processing step does not detect any additional DC but selects regions without enough signal for reliable tissue characterization. Adding the quantified acoustic shadows to the overall DC volume, could result in overestimating DC in VH because not all plaque within the acoustic shadow is calcified. However, excluding the quantified shadows from the VH results will result in larger underestimation of the DC volume, because all DC areas located in the acoustic shadow (i.e. calcifications located behind other calcified areas) will be excluded as well. Moreover, a thin or non-dense calcified plaque would allow for penetration of the acoustic signal and would likely not result in an acoustic shadow.

### Coronary artery calcium on IVUS

Coronary calcium, as assessed with different imaging modalities, is a representation of overall atherosclerotic burden. The prognostic value of coronary artery calcifications has been widely established [15]. Therefore, accurate assessment of coronary calcium on IVUS is of value in clinical practice. Calcifications on IVUS are strongly correlated with overall coronary plaque burden, but show limited correlation with stenosis severity [16].

Besides sole DC, the relation between DC and necrotic core (considered a vulnerable plaque type) is of clinical value. Previous studies addressed the prognostic value of the NC/DC ratio on IVUS VH. On IVUS VH the ratio between NC and DC was the only significant parameter associated with cardiovascular risk factors for sudden coronary death in men [17]. Moreover, the NC/DC ratio was positively associated with a high-risk acute coronary syndrome presentation [18]. Indeed, ruptured plaques have a smaller calcium arch, and relatively more deep calcium than superficial calcium compared to non-ruptured plaques [19].

The influence of coronary calcium on atherosclerotic plaque characterization with VH is an ongoing topic of debate [20]. Some studies suggest that coronary calcium on VH is surrounded by an area of artefact incorrectly quantified as NC [21]. This was confirmed by Pu et al. [22] in a study with 131 coronary lesions, combining VH with near-infrared spectroscopy (NIRS). In all lesions with calcified plaque, NC was present on VH. However, in these calcified plaques no relation was observed between percentage NC of VH and lipid core burden index on NIRS, suggesting an overestimation of NC in VH due to the artefact caused by calcium. By masking the regions behind the DC in the acoustic shadow, the median NC volume decreases compared to VH which potentially could improve the relation with NIRS. Furthermore, Thim et al. [23] found no correlation between NC size determined by IVUS VH and real histology. Although they did not include histological examples with large calcifications and acoustic shadows on the corresponding VH images, they suggest that the NC tissue detection in VH and the presence of calcifications are linked. A similar assessment of DC by VH and real histology is needed to provide further insights in this approach of enhancing VH results.

In addition to the relation with clinical presentation, coronary calcium influences the local response to medical therapy for plaque regression. Bruining et al. [24] performed IVUS in 118 patients randomized to treatment with perindopril or placebo. Patients with little calcium showed plaque regression on perindopril treatment, whereas patients with moderate calcium showed no change in atherosclerosis. This led to the concept that coronary calcium content should be considered in quantitative analysis of therapy effect in atherosclerosis regression studies and need accurate assessment.

By quantifying the acoustic shadow, the presented algorithm can enhance the assessment of DC in VH. By applying the algorithm, there is a small trade-off in the agreement of NC, but there is no significant difference in NC variance between VH and eVH as shown in the last column of Table 2. Potentially, this novel tool provides better applicability of VH for DC assessment.

### Limitations

Although results of the presented study show that the VH results can be enhanced to improve correlation and bias of DC with QCT, there are some limitations. The post-processing step was applied on VH data and analyses from a single-centre. The influence of the post-processing step on the output of different IVUS catheters/vendors is unknown, specifically if iMap [11] would benefit in the same degree of this approach. The agreement for FI and FF became less with the novel algorithm. However, for clinical purpose accurate assessment of DC and NC is of greater value. Both NC and DC on VH are associated with ACS presentation or plaque rupture, whereas FI and FF are not [1, 25].

For this comparison, the QCT was used as the gold standard. However, the segmentation of plaque areas and the tissue classification in QCT is influenced by blooming artefacts. Also, bias between both modalities is always present because of the difference in segmented plaque volumes. QCT overestimates the plaque volume by an underestimation of the lumen volume and a slight overestimation of vessel wall volume compared with IVUS [6]. A direct comparison between the plaque characterization in VH and OCT or histopathology [26] could provide further insights into the distribution of DC in areas within the acoustic shadows.

## Conclusion

Although tissue characterization within the acoustic shadow in VH is unreliable, an automatic post-processing step to quantify the acoustic shadow in order to add these regions to the calcified tissue enhances the agreement with QCT DC characterization.
